# Functional group diversity is key to Southern Ocean benthic carbon pathways

**DOI:** 10.1371/journal.pone.0179735

**Published:** 2017-06-27

**Authors:** David K. A. Barnes, Chester J. Sands

**Affiliations:** British Antarctic Survey, Natural Environment Research Council, High Cross, Cambridge, United Kingdom; Universita degli Studi di Genova, ITALY

## Abstract

High latitude benthos are globally important in terms of accumulation and storage of ocean carbon, and the feedback this is likely to have on regional warming. Understanding this ecosystem service is important but difficult because of complex taxonomic diversity, history and geography of benthic biomass. Using South Georgia as a model location (where the history and geography of benthic biology is relatively well studied) we investigated whether the composition of functional groups were critical to benthic accumulation, immobilization and burial pathway to sequestration–and also aid their study through simplification of identification. We reclassified [1], [2]) morphotype and carbon mass data to 13 functional groups, for each sample of 32 sites around the South Georgia continental shelf. We investigated the influence on carbon accumulation, immobilization and sequestration estimate by multiple factors including the compositions of functional groups. Functional groups showed high diversity within and between sites, and within and between habitat types. Carbon storage was not linked to a functional group in particular but accumulation and immobilization increased with the number of functional groups present and the presence of hard substrata. Functional groups were also important to carbon burial rate, which increased with the presence of mixed (hard and soft substrata). Functional groups showed high surrogacy for taxonomic composition and were useful for examining contrasting habitat categorization. Functional groups not only aid marine carbon storage investigation by reducing time and the need for team size and speciality, but also important to benthic carbon pathways per se. There is a distinct geography to seabed carbon storage; seabed boulder-fields are hotspots of carbon accumulation and immobilization, whilst the interface between such boulder-fields and sediments are key places for burial and sequestration.

## Introduction

The geography of carbon accumulation in biodiversity is cosmopolitan with high accumulation in rain forests, swamps, marshes, kelp forests, coral reefs And regions of up-welling, and low levels in deserts, ice sheets, ocean gyres and iceberg scoured polar shallows. The longer term pathways of immobilization and sequestration of carbon are more spatially restricted. It is becoming clearer that high latitude continental shelves may be very important to global carbon immobilization and sequestration potential. There may be a variety of reasons responsible for this such as their considerable area (1000km wide in places), depth (so are less disturbed), lower anthropogenic impact (e.g. reduced trawling frequency) and intense primary production (seasonal). Recent studies have revealed the appearance of new biological carbon sinks with ice shelf loss[3], much more rapid growth by carbon accumulators than thought possible [4]and increased carbon storage with sea ice losses[1]. If estimates from such work are correct (~ 10^7^ tonnes/year for Arctic and Antarctic shelves) the scale of benthos uptake may represent ~1% of that taken up by the Southern Ocean in buffering of anthropogenic outputs. However carbon storage by benthos may be considerably more important than previously thought for several reasons. Firstly, benthic carbon storage on very large cool temperate and sub-Antarctic shelves, such as Patagonia, Kerguelen Plateau and South-East New Zealand, have not been quantified. Secondly, frequent iceberg scour may have ‘disguised’ how highly productive Antarctica’s shallows (0–50 m) may be–with potential to immobilize ten times as much carbon [5]. Thus estimates may be revised upwards once the little studied 50–200 m depth zone is taken into account. Thirdly no account has been taken of pelagic production which is unquantified in terms of immobilization at the seabed but it could be considerable [6]. Lastly, the combination of warming surface waters[7]with increasing phytoplankton blooms [8]could increase food processing rate and food for benthos to further enhance benthic production.

Despite the importance of high latitude benthic carbon pathways there are many hurdles to a better understanding of these blue carbon ecosystem services. Complex taxonomic diversity, growth rate differences, glacial history and geography of benthic biomass are amongst the biggest challenges. Taxonomic or functional group surrogacy would reduce the current considerable requirement for taxonomic expertise across the many major animal types abundant and possibly important in the pathway from carbon accumulation to sequestration. Surrogacy has been widely geographically and taxonomically investigated with mixed results [9–11]. In the Southern Ocean it has been most examined around active fishery areas, such as South Georgia, because simplification of bycatch categories can aid CCAMLR fisheries observers to estimate and report bycatch and by doing so better regulate to reduce environmental impacts (e.g. to Vulnerable Marine Ecosystems, see [12,13]. To date it remains unclear how much a simplified classification scheme, such as functional groups (or ecological guilds) aids biodiversity assessment and conservation or represents the considerable biodiversity of polar benthos. To our knowledge it has not been applied to blue carbon assessment in the Southern Ocean or elsewhere.

Whilst most regions in the Southern Ocean have only been coarsely and patchily habitat mapped to date [14,15], South Georgia has had two more detailed contrasting seabed categorization (habitat mapping) schemes applied to it [2,16]. These two schemes differ in complexity, data sources and emphasis; [2] scheme divides the shelf into essential four categories (old sediments, young sediments, fjords and rocky moraines), uses data from two Darwin Initiative supported research cruises and is based on substrata, benthic species composition, and by proxy geological age. In contrast the [16] scheme involves more (varied) categories, using available data-based information which are physical (e.g. bathymetry and derivative data, temperature, current, salinity etc). Thus these schemes are nearly mutually exclusive in the data used, the level of spatial coverage and the scale the data is gridded at. Such variety of approaches are needed considering it is one the world’s largest Marine Protected Areas but in a hotspot region of climate change and subject to multiple stresses ([17], http://www.gov.gs/environment/marine-protected-area/). Thus within the Southern Ocean, South Georgia is an ideal model region for testing functional groups to categorise benthos for carbon storage assessments because of advantages in 1) well studied benthic biodiversity with prior functional group investigation, 2) well studied habitats with categorisation schemes, 3) contextual geological and biological estimates of seabed exposure across its continental shelf, e.g. [2,3], and [4] applied uses in fishery regulation and evaluation of threats within a Marine Protected Area. Our first hypothesis is that functional groups are important to the benthic carbon pathway, but that the geography and nature of substratum will also be important (previous work has shown biodiversity is most linked with old boulder fields and to a lesser extent old sediments,[2]).Our second is that there can be reasonable taxonomic surrogacy by benthic functional groups (providing there is enough knowledge of the diversity of benthos to assign morphotypes to meaningful functional groups and enough groups are used). We also test contrasting published habitat mapping schemes and attempt to erect a schematic map of seabed carbon pathway importance, to serve as a future hypothesis testing tool.

## Materials and methods

The fieldwork was carried out during two Darwin Initiative funded scientific cruises in 2011 (JR262) and 2013 (JR287) of the RRS James Clark Ross to the isolated continental shelf around South Georgia, in the Southern Ocean. Permission for scientific work and seabed sampling within South Georgia’s coastal waters was given by the Government of South Georgia and South Sandwich Islands, as part of their collaboration with Darwin Initiative projects 18–019 and EIDCF013. The Polar Front flows eastwards 200km to the north of South Georgia’s shelf, whilst the Southern Antarctic Circumpolar Current Front flows around the eastern shelf anticlockwise from SE to NE. Regional current velocities and directions have been modelled but not at a scale in time or space likely to be relevant to benthos. Although there have been water column mass flux estimates for the broad region, we know of no literature that could differentiate between sites within the shelf, and likewise for sediment carbon content.

**Fig 1:**
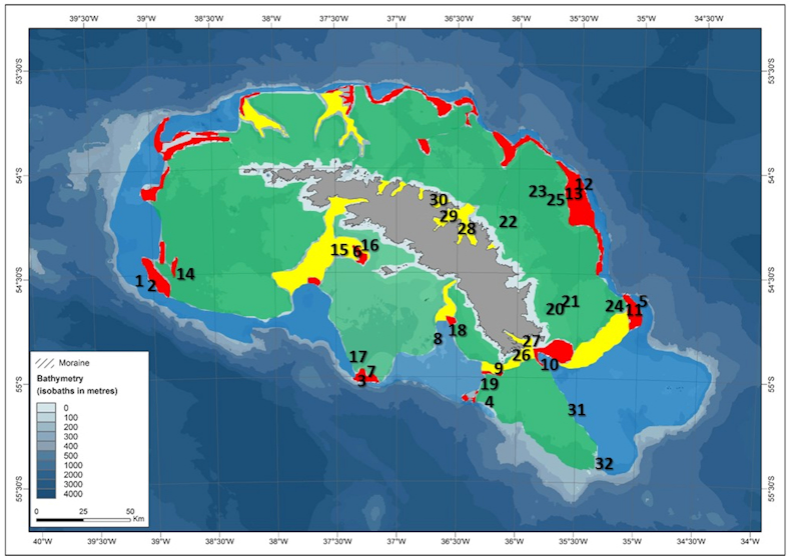
The Southern Ocean continental shelf around South Georgia, with study sites and major habitat categories of Barnes *et al*. (2016b). The habitats are old, outer sediments (blue), young basin sediments (green), fjord and canyons (yellow) and moraines (red).

Details of the 32 study sites and sample apparatus are given in [2], which attempted to survey the continental shelf from shelf break to coast, across habitat types and depths (85–322 m)(Fig 1). Samples consisted of 67 trawls (approximately 100kg wet mass of benthos) taken with a 2 x 0.5 m Agassiz trawl, towed for 5 minutes at 0.5 knots. The other apparatus used was the Shelf Underwater Camera System (SUCS). We made 30 SUCS deployments, each of which yielded 20 high resolution, quantitative images of seabed and benthos. Functional group characterization was targeted around factors that we considered important to carbon pathway potential. We tried to categorize benthos recorded into the minimum number of functional groups without combining morpho-species of differing feeding types, mobility and skeletisation (Table 1). Four values of benthic carbon storage were considered from the South Georgia sites; two from literature (accumulation [1], and immobilization [18]), one observed from images (burial rate) and one estimated (sequestration–long term storage of buried carbon). Samples were dried for 48 hr at 70°C and weighed to obtain dry mass and then further ashed at 480°C and reweighed to obtain ash-free dry (organic) mass. We defined carbon accumulation as the carbon proportion of dry mass (following [18]). Carbon was only considered immobilized in the subset of calcareous skeletonized animals. In these we multiplying ash free dry mass bound within the skeleton by 0.5 [19] and adding the value to the carbon proportion of skeletal mass which we calculated to be approximately 13.3% (±2.5%). Typical groups with high immobilized carbon levels were bryozoans, corals, hydrocorals, calcareous polychaetes and sponges, as well as bivalve and brachiopod shells) from [2]. Frequency of partial burial of benthos was analysed from -SUCS images taken at each site. These images were all exactly perpendicular to substratum, taken at the same distance, aperture and magnification, and the field of coverage has been *a priori* calibrated for error (e.g. differential distortion from centre to outer edge of lens).We estimated sequestration potential using immobilization data, partial burial data, sediment proximity and literature [20] for each site.

**Table 1:** Functional group categorization of benthos on South Georgia’s shelf.

Functional group	Example taxa
Pioneer sessile suspension feeders	Encrusting bryozoans, ascidians, some polychaetes
Climax sessile suspension feeders	Demosponges, glass sponges, brachiopods
Sedentary suspension feeders	Basket stars, valviferan isopods, some polychaetes
Mobile suspension feeders	Some brittle stars, crinoids, krill
Epifaunal deposit feeders	Sea cucumbers, some polychaetes
Infaunal soft bodied deposit feeders	Some polychaetes, echiurans, sipunculans
Infaunal shelled deposit feeders	Bivalves, irregular sea urchins
Grazers	Regular sea urchins, limpets
Soft bodied, sessile scavenger/predators	Sea pens, soft corals, anemones, hydroids
Hard bodied, sessile scavenger/predators	Cup corals, whip corals, hydrocorals
Soft bodied, mobile scavenger/predators	Some polychaetes, nemerteans, octopus
Hard bodied, mobile scavenger/predators	Sea stars, fish, gastropods, some brittlestars
Jointed legged, mobile scavenger/predators	Sea spiders, shrimps, amphipods

We used two main techniques for analysis. To assess significance of potential factors we used ANOVA and regression, whilst to investigate taxon surrogacy and habitat categorization we used non-metric multidimensional scaling (nMDS) ordination, using the VEGAN package in the statistics software R. We calculated and plotted the geography of density (of benthic individuals; ind/m^2^) and richness (of species; no. species/site) by site, followed by the number of functional groups present (no. functional groups/site) and which functional group was most represented. The factors included in analyses were, by site, the number of functional groups present, the proportion of sessile suspension feeders (because they were well represented at most sites with high carbon storage values), the number of morpho-species (richness), trophic levels, size spectra (how many orders of magnitude), morpho-species categorized under CCAMLR as ‘vulnerable marine ecosystem’ and rare morpho-species (one-two total occurrences) present. The substratum of each image from each site was categorized as hard (boulder field), soft (sediment) or mixed (boulders and sediment). No images showed bedrock. Carbon accumulation, immobolization and sequestration estimate data were all log transformed in order to regress (linear) lines of best fit to proportion of substratum types. We performed nMDS ordinations using two data sets; The first data set consisted of functional group abundance data only. This was shown in two dimensional plots, with site points sequentially coloured by habitat categories from [2] and then [16] for comparison. These were compared with a similar nMDS using morphospecies (rather than functional groups) from the same sites, to assess taxon surrogacy of functional groups. The second data set used for nMDS was wider incorporating all measured potential factors (from ANOVA), as well as functional groups.

We consider the relative contribution of carbon accumulation, immobilisation, sequestration (estimate), conversion rate of accumulation to immobilization and conversion from immobilization to sequestration to each habitat site. Each of the five factors were tested between sites using the Kruskal-Wallis rank sum test implemented in R. The mean carbon pathway importance was metricised (from the sum of ranks from the carbon measures). This was tested for fit against both [2]and [16] habitat categorization schemes. The ranked overall carbon pathway importance was compared across habitat types using Kruskal-Wallis rank sum test, with multiple pair-wise post hoc Tukey tests. Finally the carbon pathway data by site was used to construct a georeferenced schematic of geographic zonation of benthic carbon importance. The main purpose of this schematic was an attempt to geographically simplify the ecosystem service of carbon storage as a testable framework for future biological and geological sampling.

## Results

**Fig 2:**
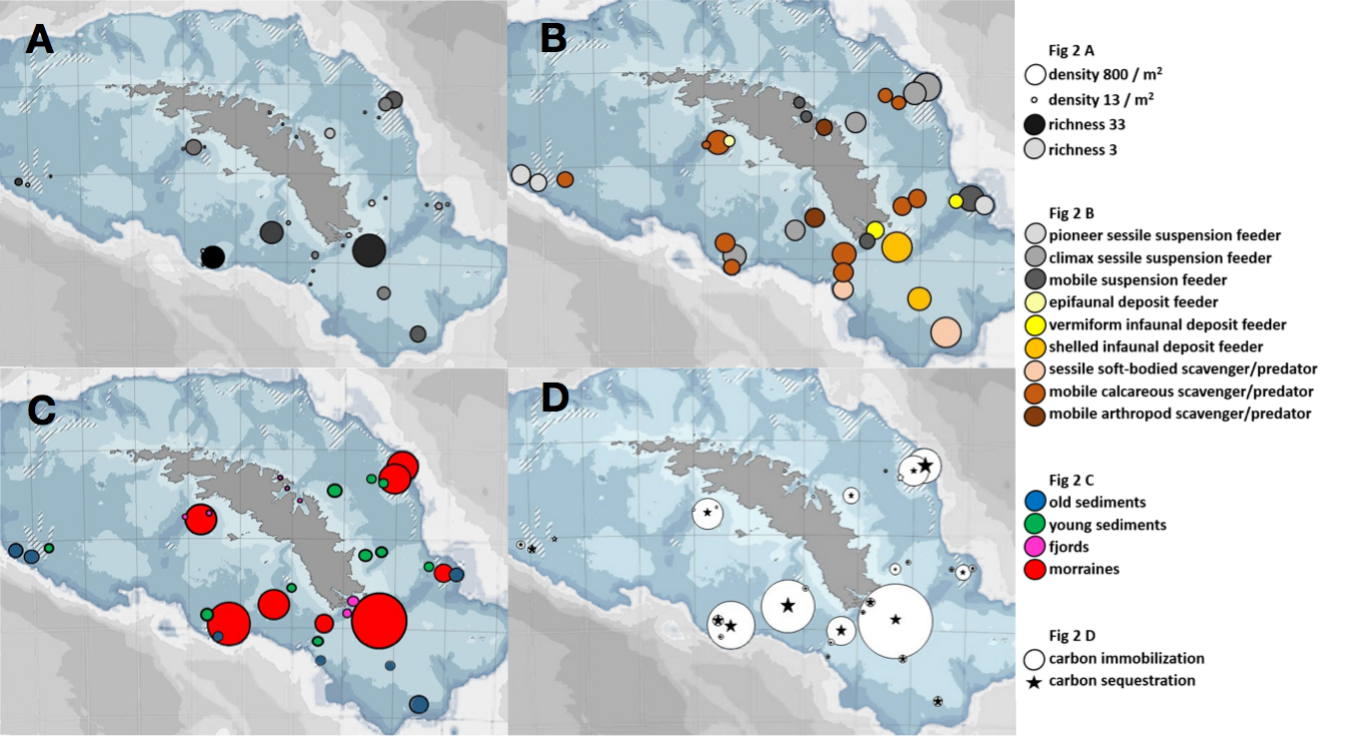
Measures of benthic colonization, seabed carbon stocks and functional group partitioning at South Georgia, Southern Ocean. The plots are benthos density and richness (A) at sample sites, in which the size of point increases with density and the darkness of point increases with richness. Functional group diversity (B) in which size increases with number of functional groups and colour represents which functional group is numerically dominant. Carbon accumulation in benthos (C) in which size increases with C magnitude and the colours represent habitat category of Fig 1. Carbon immobilization and estimate of sequestration (D) in which symbol size increases with magnitude of C immobilization (circles) and sequestration estimate (stars). All data are given in supplementary materials S1 Table.

The minimum number of benthos categories that we considered possible for our South Georgia benthos data, in terms of carbon storage, was 13 functional groups (Table 1). Density and richness varied considerably (Fig 2A) both between sites, and within and between habitats (see Fig 1). Likewise the number of functional groups and the dominant functional group also varied between sites, and within and between habitats (Fig 2B). The least ubiquitous functional group were the grazers (such as the regular echinoid *Sterechinus*), present at just six sites whereas the most ubiquitous were hard-bodied mobile scavenger predators (such as the brittle star *Astrotoma*) present at 31 sites. The latter functional group was also the most numerous overall whilst the epifaunal deposit feeders (such as the holothurian *Psolus*) were the least abundant. Functional group diversity was high, with 4–12 present at any one site and 7 different functional groups dominated across sites. No one functional group dominated all sites in any habitat type, some functional groups dominated at sites across contrasting habitats and some did not dominate at any sites. Values of carbon accumulation (Fig 2C) and immobilization (Fig 2D circle symbols) were highest at boulder field sites (coloured red). However the highest conversion rates of accumulation into immobilization occurred at sites 9, 18 and 31 which represented three differing habitats. Mean conversion rate of carbon of accumulation into immobilization was just less than 20%. High carbon sequestration estimate were more numerous (than sites of high accumulation and sequestration) and spread across more habitats (Fig 2D star symbols). Mean conversion rates of immobilization to sequestration were about 34% but this value was boosted at some sites (14, 25 and 2) from external input [these were not treated differently in analyses however]. These sediment sites were downslope from high carbon immobilization sites, in which we saw biological material (broken bryozoan skeletons, worm tubes and coral) which had probably cascaded in from nearby sites increasing chances of burial and sequestration.

**Fig 3:**
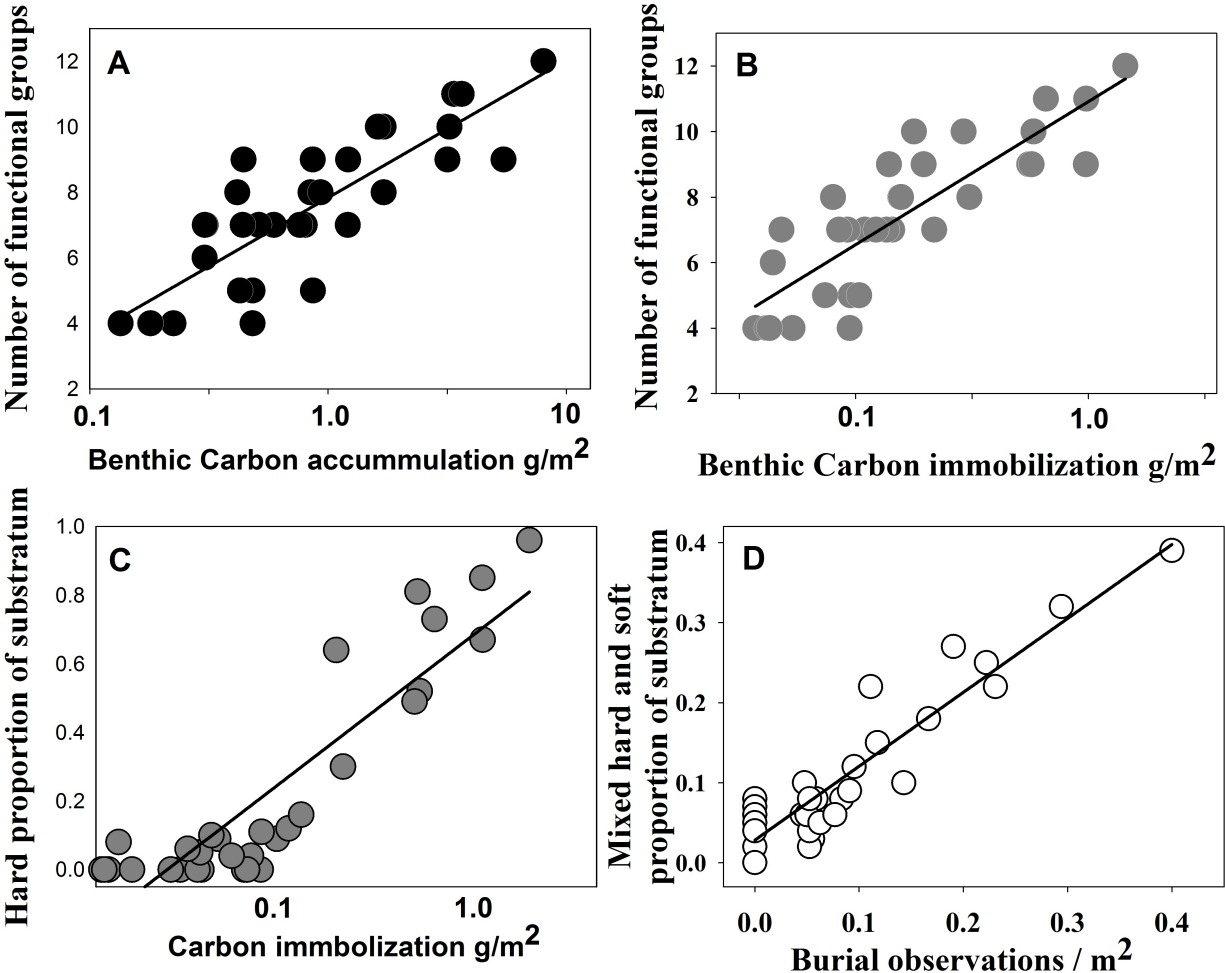
Benthic carbon, functional groups and substratum relationships at South Georgia. Increase in carbon accumulation and immobilization with number of benthos functional groups (A and B respectively). Carbon immobilization with the proportion of substratum which is hard (boulder and cobble rubble) (C). Number of benthos part burial observations with the proportion of substratum which is mixed (boulder and cobbles with mud) (D). The associated ANOVA statistics are F = 84.6, 85.8, 69.6 and 153.1 for Fig 3A-D respectively, all p<0.001.

The ANOVA results showed that number of functional groups present was by far the most significant factor in carbon accumulation (Table 2) and the only significant factor in carbon immobilization (Table 3) and sequestration estimate (Table 4). In contrast, no benthos characteristic emerged as a significant factor in [carbon] burial rate, including functional groups (Table 5).The relationship between functional group number and carbon accumulation (Fig 3A) and immobilization (Fig 3B) represented 79% and 83% of variability in logged data. Carbon immobilization also increased with increasing presence of boulder field hard substrata (Fig 3C)–note that immobilization values were approximately an order of magnitude lower than carbon accumulation values. Few partial burials of benthos were observed in boulder field or sediment images but burials increased with presence of mixed (both hard and soft) substrata (Fig 3D).

**Table 2:** Carbonaccumulation across macrobenthic functional groups at South Georgia. The values are GLM ANOVA output, with most significant factor shown in bold. *P* values are shown (*P< 0.05 and **P< 0.01).

Source of variation	*df*	*Adj SS*	*Adj MS*	*F ratio*	*P*
**No. Functional Groups**	**8**	**13.2360**	**1.65450**	**4.09**	0.007**
% Suspension feeders	1	2.2766	2.2765	5.63	0.030*
Richness	1	1.0516	1.0516	2.6	0.125
Trophic groups	1	0.0947	0.0947	0.23	0.635
Size	1	0.1311	0.1311	0.32	0.576
VME	1	0.0212	0.0212	0.05	0.821
Rarity	1	0.0013	0.0013	0.00	0.956
Error	17	6.8742	0.4044		
Total	31	94.028			

**Table 3:** Carbonimmobilisation across macrobenthic functional groups at South Georgia. The values are GLM ANOVA output, with most significant factor shown in bold. *P* values are shown (*P< 0.05 and **P< 0.01).

Source of variation	*df*	*Adj SS*	*Adj MS*	*F ratio*	*P*
**No. Functional Groups**	**8**	**0.2819**	**0.0352**	**3.88**	0.009**
% Suspension feeders	1	0.0056	0.0056	0.61	0.445
Richness	1	0.0305	0.0305	3.36	0.084
Trophic groups	1	0.0065	0.0065	0.71	0.410
Size	1	0.0079	0.0079	0.87	0.365
VME	1	0.0452	0.0452	4.98	0.039*
Rarity	1	0.0010	0.0010	0.11	0.740
Error	17	0.1543	0.0091		
Total	31	3.5081			

**Table 4:** Carbonsequestration across macrobenthic functional groups at South Georgia. The values are GLM ANOVA output, with most significant factor shown in bold. *P* values are shown (*P< 0.05 and **P< 0.01).

Source of variation	*df*	*Adj SS*	*Adj MS*	*F ratio*	*P*
**No. Functional Groups**	**8**	**0.0496**	**0.0063**	**3.99**	0.008**
% Suspension feeders	1	0.0037	0.0037	2.40	0.140
Richness	1	0.0002	0.0002	0.15	0.704
Trophic groups	1	0.0001	0.0001	0.02	0.895
Size	1	0.0008	0.0008	0.50	0.489
VME	1	0.0001	0.0001	0.01	0.943
Rarity	1	0.0009	0.0009	0.56	0.290
Error	17	0.0264	0.0016		
Total	31	0.2103			

**Table 5:** Carbonburial across macrobenthic functional groups at South Georgia. The values are GLM ANOVA output, with most significant factor shown in bold. *P* values are shown (*P< 0.05 and **P< 0.01).

Source of variation	*df*	*Adj SS*	*Adj MS*	*F ratio*	*P*
No. Functional Groups	8	0.0516	0.0645	0.56	0.799
% Suspension feeders	1	0.0061	0.0061	0.52	0.479
Richness	1	0.0008	0.0008	0.08	0.786
Trophic groups	1	0.0035	0.0035	0.30	0.591
Size	1	0.0131	0.0131	1.13	0.303
VME	1	0.0045	0.0045	0.39	0.543
Rarity	1	0.0043	0.0043	0.37	0.549
Error	17	0.1976	0.0116		
Total	31	0.2878			

**Fig 4:**
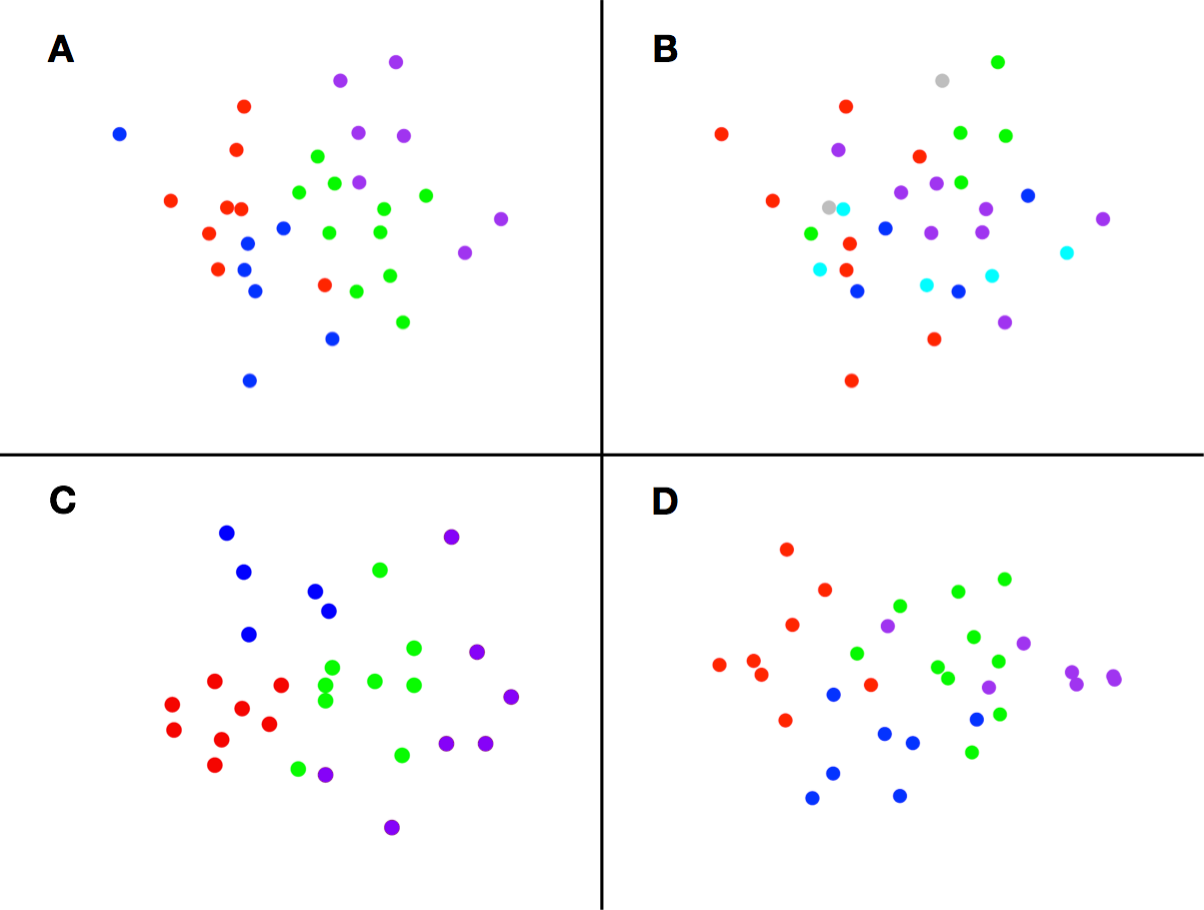
Non-metric multidimensional scaling (nMDS) ordination of benthos using different habitat and benthos categories. Each point represents a site from Fig 1. Benthos ordinated by functional groups and displayed in Barnes *et al*. (2016b) habitat categories (colours in Fig 1)(A). Benthos ordinated by functional groups and displayed in Hogg *et al*. (2016) habitat categories (B). Benthos ordinated by morphospecies in habitat categories from Barnes *et al*. (2016q) (C). Benthos ordinated by functional groups, carbon storage and biodiversity characteristics and displayed in Barnes *et al*. (2016q) habitat categories (D).

Ordination (nMDS) of functional group composition by site did not show distinct clusters (Fig 4A). However for the most part sites within-habitat categories of [2]grouped closest to each other (colours of Fig 4A). Although there was overlap between moraine boulder field (coloured red) and outer old sediments (blue) in Fig 4A, the pattern in our functional group data much more closely aligned to habitats than in an alternative habitat scheme (Fig 4B). Strong taxon surrogacy was demonstrated as the degree of separation between habitats and level of dispersion within each habitat in Fig 4A showed close similarity to that using morpho-species data (Fig 4C). Reordination of site data using additional wider biological characters (carbon accumulation, immobilization and sequestration estimate, richness, trophic levels, size spectra, VMEs, and rarity) showed a similar pattern, but with less separation of fjordic/canyon and cross shelf sediment habitats (Fig 4D).

No clear pattern of carbon pathway importance was found across the sites, although [2]habitat categories of moraine and fjord significantly differed in terms of carbon accumulation (Kruskal-Wallis rank sum test, χ_df = 3_20.2, p<0.001), immobilisation (KW: χ_df = 3_ = 21.6, p<0.001), sequestration (KW: χ_df = 3_ = 16.9, p<0.001), immobilisation conversion to sequenstration (KW: χ_df = 3_ = 12.5, p<0.01) and overall ranked carbon values (KW: χ_df = 3_ = 17.5, p<0.001). Post Hoc Tukey tests showed moraine rubble habitats had significantly higher (all p<0.05) carbon storage than any other habitat. The sites with lowest carbon pathway values were all in fjord and canyon systems, but Drygalski fjord (sites 26 and 27) had moderate to high values. Generally the sites of most carbon pathway importance were found around moraine boulder fields, but not always. Carbon pathway importance was a better fit to the [2]habitat categories than those of [16] but neither were a strong fit. Functional groups may show a clear pattern and have a strong relationship with carbon pathway but we could not detect a straight-forward link between benthic carbon attributes and existing habitat schemes. Overall our results suggest measurement of functional group richness is the easiest and most powerful way to assess regional blue carbon importance.

## Discussion

For its size and remoteness South Georgia is well sampled and studied, it’s marine biodiversity abundant, rich and highly endemic, and the associated fishery, tourism industry and marine protected area all tightly and effectively regulated (see [21] and http://www.gov.gs/environment/marine-protected-area/).This robust contextual background means South Georgia offers one of the best possibilities within the polar regions for meaningful assessments of benthos carbon storage possibilities within a discrete area (i.e. the continental shelf does not link to those around continent margins). Benthic carbon cycling begun to be investigated in the North Atlantic and Pacific three decades ago, e.g. [22], establishing just how important macro and mega benthos were, particularly with respect to storage. In the Arctic, there has been significant recent progress in terms of measurement, analysis and modelling of carbon cycling and pathways [23,24]. However such work shows how complex, even just one element of blue carbon, such as benthos pathways are. This complexity, combined with the increasing Arctic and Antarctic carbon sinks in response to climate-forced sea ice losses [1,25], shows the importance of establishing and ground-truthing methods to simplify measurement, analysis and monitoring this valuable ecosystem service. The combination of geological [26,27,28] and biological [2] evidence streams should effective tools as constraining error in reconstructing glaciation histories, and thereby climate modelling. The current study evaluated functional groups of benthos to reduce identification effort, time and expertise but also recognition of areas of different carbon importance through habitat categorization e.g. [2,16].

### Importance of functional groups

Use of functional traits and groups to understand environment processes such as nutrient cycling has been widespread across terrestrial and aquatic environments [29–31]. Their use in marine ecology has been more frugal but see[32,33] especially in polar environments. However it has proved highly successful, especially for examining nutrient cycling in sediment macro fauna e.g. [34]. Using such an approach would seem ideal for examining carbon pathways amongst the very rich benthic biodiversity found on Southern Ocean continental shelves (http://www.scarmarbin.be/). In the current study the importance of functional groups extended beyond mere simplification of complexity. Functional groups are, at South Georgia at least, clearly important to benthic carbon storage pathways, in terms of both number (Tables 2–4, Fig 3A and 3B) and composition (Fig 4A). However quite why and how this was the case has some obscure elements to it, such as neither the presence nor absence of any one functional group seemed critical to any of the carbon pathways (e.g. accumulation). It seems that relationships between seabed carbon storage and benthic functional groups are complex (Fig 2C), influenced by abiotic factors such as substratum (Fig 3C and 3D). Also there was no significant relationship between functional groups and the critical pathway stage of burial (Table 5). The first hypothesis, that functional groups are important to benthic carbon pathway, is accepted as is the nature of substratum (particularly to burial) but key questions remain; 1) why is it that the number of functional groups is so important to carbon storage, or is this merely correlating with an underlying factor not yet elucidated? One possible answer is that as functional group richness reflects taxonomic group richness, which is likely to correlate strongly with length of time undisturbed. 2) what makes some sediment sites very much more important to carbon pathways than others. A possible answer to this may be the length of time since last ice scour, which is certainly a powerful explanatory variable in the shallows [35]. More comprehensive soft substratum sampling, for example with a multicore, and examination of factors such as grain size, organic and oxygen content should shed some light on at least the second question.

### Surrogacy and functional groups

Our South Georgia data show that benthic functional groups and habitats can be strong surrogates for morpho species level patterns (Fig 4A vs 4C). Both in terrestrial [10] and marine ecology [9,11] functional groups and habitats have been widely trialled to aid understanding of environment processes, management and conservation. Use of such a technique to investigate ecosystem services of carbon capture and storage, is as far as we know, relatively novel. The major advantages of using functional groups are clearly that such a method can potentially give massive gains in reducing environment assessment cost and time, but at the disadvantage of loss of resolution. The similarity of output in Fig 4A and 4C shows that functional groups can closely reflect those using morpho species taxonomic breakdown. Likewise the close grouping by colour in each of Fig 4A and 4C shows that habitat can also be an effective surrogate in benthos, but the lack of colour proximity in Fig 4B shows that it depends what habitat scheme is used. We found habitats to be significant surrogates for carbon storage (Kruskal-Wallis rank sum tests), but ordination (Fig 4D) showed little evidence for separation of fjordic and inner shelf sediments. Overall we accept our second hypothesis that there is reasonable taxonomic surrogacy by benthic functional groups and habitats but suggest that neither existing habitat scheme [2,16] for our example location, South Georgia, is ideal.

### Polar blue carbon assessments

Aside from historic and future harvesting of living resources, one of the significant societal benefits from life in the Southern Ocean is the provision of blue carbon ecosystem services. Furthermore it seems to be increasing in magnitude with regional warming, and unlike further south such gains are not nullified by iceberg scour [5]. However the current study suggests that there is not an easy mechanism for monitoring seabed blue carbon performance. If the benthic carbon values on South Georgia continental shelves are representative of wider Southern Ocean patterns then both the biology and geography of carbon pathways is more complex than envisaged. Neither hot-spots nor cold-spots of benthic macrofaunal carbon map straight-forwardly on to existing habitat schemes of[2,16]or onto the possession or absence of particular functional groups. The highest values of benthic carbon accumulation and immobilization were all linked with rubble habitat (left from the Last Glacial Maximum in moraines, see [28]). This is intuitive as many large ‘habitat forming’ bioconstructors, such as sponges and corals require hard substratum to establish and anchor–they then facilitate increased growth of other benthos. In contrast the lowest values were all associated with coastal fjords, but some fjordic sites (e.g. site 27) had high values and some shelf sediments had higher carbon accumulation than some rubble sites (e.g. sites 32 and 22). We think this may on part be explained by the occurrence of fish, which are considerable carbon accumulators but immobilise little of this, as on death most of their carbon is quickly recycled (e.g. by scavenging, [36]). An ypattern is more obscure in terms of carbon sequestration, apart from most coastal fjordic sites being low–yet this locking away of carbon is arguably (at least from an anthropogenic perspective) the most important component of the pathway. No pattern was apparent to the ranking of sites by conversion rate of carbon accumulation to immobilization either in terms of particular functional group presence or absence, or habitat nature. However the highest conversion rates of carbon immobilization to sequestration were associated with sediment near moraine rubble (e.g. sites 14, 25, 2 and 17). This probably reflects the combination of proximity to carbon immobilization hotspots (Fig 2D) and high burial rates where hard and soft substrata meet (Fig 3D). The ‘cold-spots’ of lowest conversion rates of carbon immobilization to sequestration were associated with either fjords or rubble, presumably because little carbon is immobilized in fjords to bury and although substantial carbon is immobilised on rubble, there is little associated sediment to bury it. We did find a significant fit of [2]to overall carbon pathway importance (overall ranked carbon values, KW: χ_df = 3_ = 17.5, p<0.001) but it is clear that optimal conditions for accumulation and immobilization are not those for conversion rates or sequestration.

### Polar blue carbon data cold-spots

**Fig 5:**
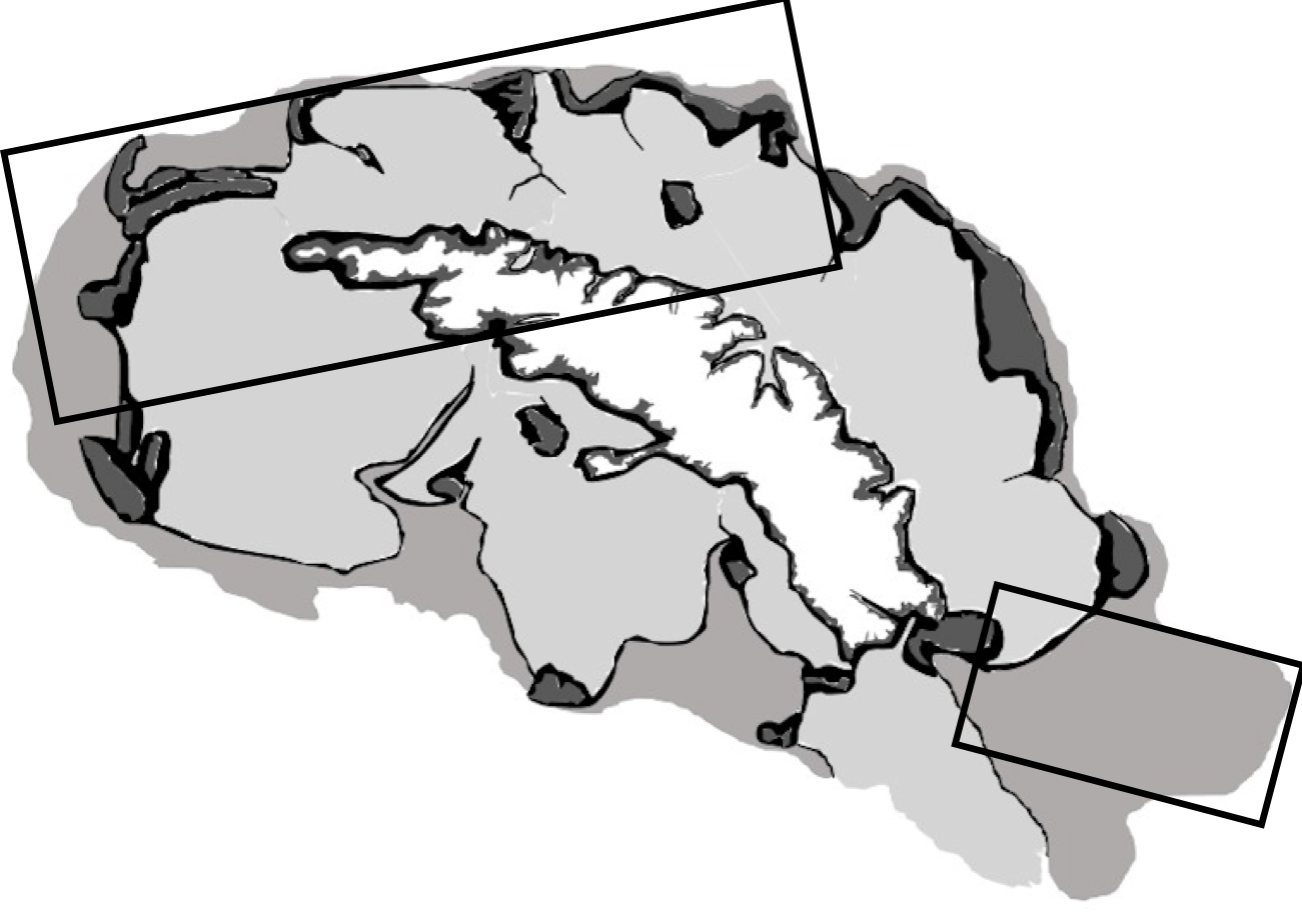
Schematic estimating different carbon storage zones across South Georgia’s continental shelf. The shades are from lightest grey (low benthos carbon), mid grey (moderate benthos carbon), dark grey (high carbon immobilization but low conversion to sequestration) and black (moderate carbon immobilization but high conversion to sequestration). The black box outlines indicate areas that were not sampled with respect to benthos Carbon characteristics.

At South Georgia the areas least sampled in terms of data from which carbon pathway values can be extracted are the Northwest and East northeast (boxes on Fig 5). For the Northwest this is surprising in that it is one of the most data rich areas for species records, reflecting its fishery importance see[23]. New information from the NW cold-spot could be gained from the Government of South Georgia and South Sandwich Islands plan to sample there in order to monitor the effectiveness of ‘closed areas’ (compared with similar adjacent areas worked by long line fisheries). Likewise the East northeast cold-spot could be reduced by sampling planned by the Antarctic Seabed Carbon Capture Change project of the Swiss-led Antarctic Circumnavigation Expedition (of 2016/17). Elsewhere we would suggest the key locations to sample are the southern Patagonian shelf, the Kerguelen Plateau and the south New Zealand shelf around the Auckland, Campbell and Antipodes islands–simply because these three areas dominate the ice-free southern, high latitude, continental shelf area. Although their shelf area is small, the many isolated sub-Antarctic archipelagos (such as Bouvetoya, Prince Edward Islands, Iles Crozet etc) represent a valuable opportunity to understand the complexity of benthic carbon pathways. This is because they potentially represent a great variety of discrete but different benthic conditions and thus may be a natural laboratory for exploring blue carbon potential, and by coring old sediments, perhaps past responses to warming at the end of previous glaciations.

## Supporting information

S1 Table All data in used in these analyses. (XLSX)
